# Multi-omics reveals mechanisms of resistance to potato root infection by *Spongospora subterranea*

**DOI:** 10.1038/s41598-022-14606-y

**Published:** 2022-06-25

**Authors:** Sadegh Balotf, Richard Wilson, David S. Nichols, Robert S. Tegg, Calum R. Wilson

**Affiliations:** 1 Tasmanian Institute of Agriculture, New Town Research Laboratories, University of Tasmania, 13 St Johns Avenue, New Town, TAS 7008 Australia; 2 Central Science Laboratory, University of Tasmania, Hobart, TAS 7001 Australia

**Keywords:** Computational biology and bioinformatics, Microbiology, Molecular biology, Plant sciences

## Abstract

The pathogen *Spongospora subterranea* infects potato roots and developing tubers resulting in tuber yield and quality losses. Currently, there are no fully effective treatments for disease control. Host resistance is an important tool in disease management and understanding the molecular mechanisms of defence responses in roots of potato plants is required for the breeding of novel resistant cultivars. Here, we integrated transcriptomic and proteomic datasets to uncover these mechanisms underlying *S. subterranea* resistance in potato roots. This multi-omics approach identified upregulation of glutathione metabolism at the levels of RNA and protein in the resistant cultivar but not in the susceptible cultivar. Upregulation of the lignin metabolic process, which is an important component of plant defence, was also specific to the resistant cultivar at the transcriptome level. In addition, the inositol phosphate pathway was upregulated in the susceptible cultivar but downregulated in the resistant cultivar in response to *S. subterranea* infection. We provide large-scale multi-omics data of *Spongospora*-potato interaction and suggest an important role of glutathione metabolism in disease resistance.

## Introduction

Potato (*Solanum tuberosum*) is the world’s third most important food crop for human consumption^1^. Root and tuber infection by soil-borne pathogens is a constant threat to potato production, resulting in considerable yield and quality losses^2,3^. The infection of plants by pathogens will results in induced responses measurable at the transcriptomic, proteomic and metabolomic levels^4^. During infection, pathogens attempt to change the host environment in their favour to facilitate colonisation and propagation. Plants, however, have developed several defence mechanisms to resist pathogen invasion^5^. Using specific cell surface receptors, plants recognise microbe-associated molecular patterns (MAMPs) which induce a cascade of defence responses to quell microbial attack. In response, pathogens can suppress the plant MAMP-triggered immunity by delivering effector proteins into the plant cell. Plants may in turn possess resistance (R) genes that recognise effectors and induce a secondary defence cascade (effector-triggered immunity). Plant defence responses will include the accumulation of reactive oxygen species (ROS), pathogenesis-related proteins, plant hormones and defensive secondary metabolites^6^. For example, in the interaction between barley and the biotrophic pathogen *Ascomycota phylum* (powdery mildew), production of ROS is the major factor in the host^7^. The induction of ubiquitous glutathione S-transferases (GSTs) have been reported in fungal, bacterial and viral infections^8^. GSTs are multifunctional enzymes associated with plant biotic stress, the upregulation of GSTs in response to pathogen infection can be related to their detoxification and antioxidant activity and the silencing or overexpression of some GST genes can modify pathogen multiplication rates and disease symptoms. However, very little is known about the exact metabolic functions of disease-induced GST enzymes^9^. Lignification and cell wall thickness are further barriers to prevent penetration of pathogens and restrict the growth of pathogens^10,11^.

*Spongospora subterranea* is an economically important soil-borne pathogen of potato world-wide^12^. Tuber surface lesions and root galling are typical visual symptoms of potato infection by *S. subterranea*^13^. The thick-walled dormant spores of the pathogen form within both root galls and tuber lesions^14^. Currently, there are no fully effective treatments for the control of *S. subterranea* disease^15^. One of the most promising approaches to combat *S. subterranea* infection is through breeding resistant potato cultivars. The introgression of *S. subterranea* disease resistance genes require the understanding of the inducible defense responses in roots of potato plants, particularly during colonisation of the root by *S. subterranea*. However, the interaction between a soil-borne biotrophic pathogen such as *S. subterranea* and its host plant is poorly understood.

Understanding the dynamic regulatory network of plant-pathogen interaction is a major challenge for developing a new disease management strategy as well as the improvement of yield and quality of crops. Plant response to biotic stress is a multi-dimensional and complex process. To reveal the regulatory mechanism of plant-pathogen interaction, multiple omics (multi-omics) approaches are required^4^. In this study we examined the response of disease resistant and susceptible potato cultivars to *S. subterranea* infection using a combination of transcriptomics and proteomics that shed light on the cellular processes involved in disease resistance, including alterations to glutathione metabolism and cell wall structure.

## Results

### Root infection by *S. subterranea* induced general defence responses in both resistant and susceptible cultivars in transcriptome level

**Figure 1:**
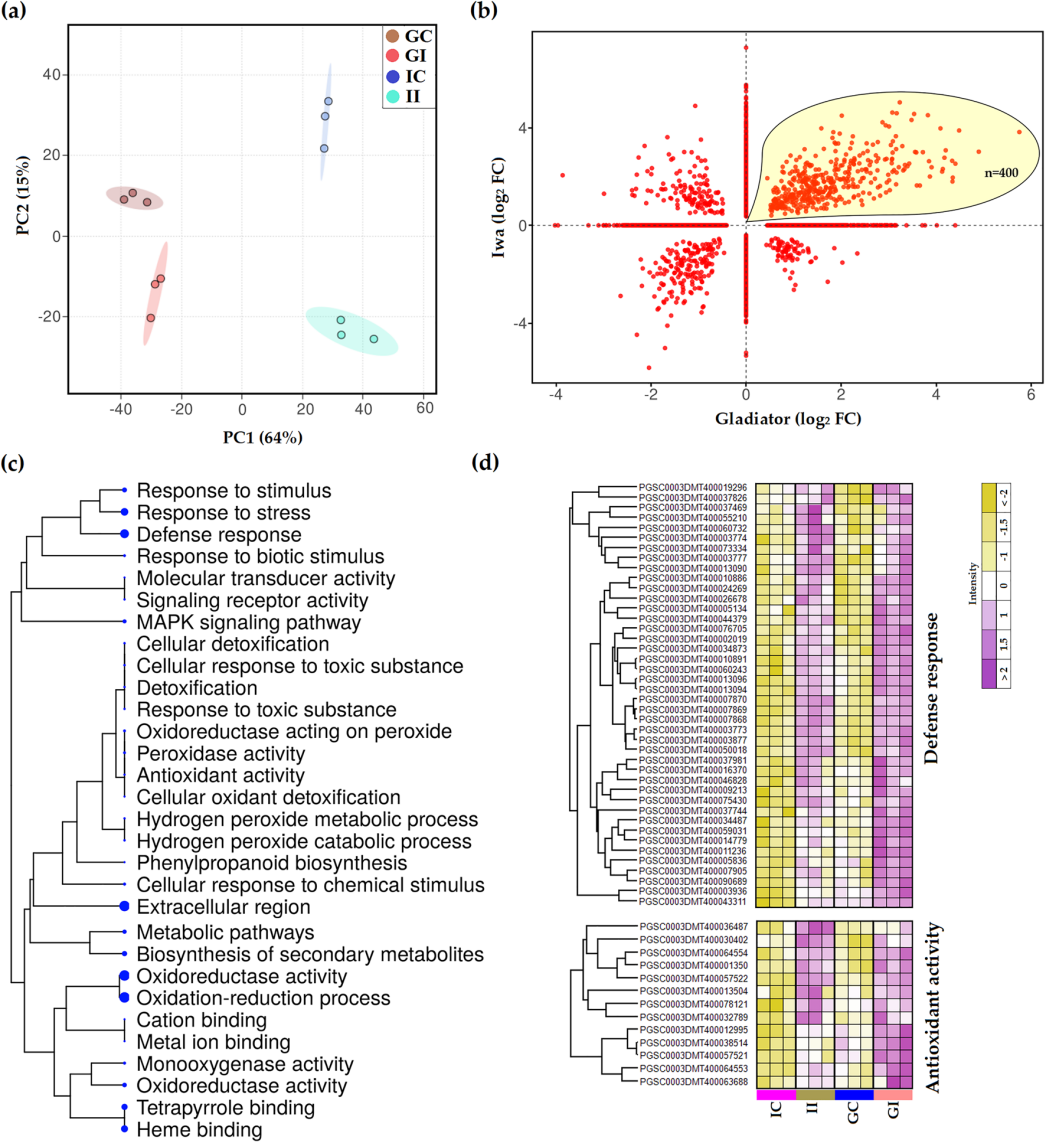
Transcriptional responses of potato to *S. subterranea* infection. (**a**) Principal component analysis (PCA) differentiates the transcriptomes of control and treated samples from Gladiator (resistant cultivar) and Iwa (susceptible cultivar). (**b**) Distribution of differentially expressed genes (DEGs) common between Gladiator and Iwa. The highlighted area shows that a total of 400 genes were significantly upregulated in both Gladiator and Iwa. The changes in genes were presented as log-transformed fold changes. (**c**) A hierarchical clustering tree summarising the correlation among significant pathways of the upregulated DEGs common between Gladiator and Iwa. Pathways with several shared genes are clustered together. Bigger dots (blue dots) indicate more significant *P*-values. (**d**) The Z-scored (normalized) expression of defence response and antioxidant activity genes. GI, Gladiator infected; GC, Gladiator control; II, Iwa infected; IC, Iwa control.

In this study, we used multiple omics approaches to characterize the response to persistent stress caused by *S. subterranea* infection in resistant (Gladiator) and susceptible (Iwa) potato cultivars. Therefore, a tissue sampling timepoint at which both cultivars showed symptoms of *S. subterranea* infection (Fig. S1) was chosen (42 days post-inoculation). Deep RNA sequencing was first used to detect contrasting gene expression patterns between the resistant and susceptible potato cultivars infected by *S. subterranea*. This RNA-seq approach resulted in over 47 million mapped reads (to potato genome) on average per treatment (Fig. S2). According to PCA, the cultivar differences accounted for the largest observed variance in transcriptomes of potato roots (PC1 > 60%) (Fig. 1a). Within the cultivars, the difference between infected and control (uninfected) samples was more marked for the Iwa transcriptome compared with the Gladiator transcriptome. This suggested that the extent of the transcriptional adjustment to *S. subterranea* infection is greater in Iwa than in Gladiator. We found 2616 genes in Gladiator (FDR < 5%) and 3887 genes in Iwa (FDR < 5%) that were differentially expressed in response to *S. subterranea* infection (Fig. S3). The log_2_ transformed fold-differences for the differentially expressed genes (DEGs) in both cultivars were displayed using in a two-way plot (Fig. 1b). The highlighted area shows that a total of 400 genes were significantly upregulated in both Gladiator and Iwa. Functional enrichment analysis of these genes identified a number of significant processes including defence response, detoxification, response to biotic stimulus, response to stress and antioxidant activity (Fig. 1c). This data showed that *S. subterranea* infection induced defence responses in both resistant and susceptible cultivars. The individual expression of the DEGs that were upregulated in both cultivars and were involved in the plant defence response (n = 42) and antioxidant activity (n = 13) is presented in a heatmap (Fig. 1d). In both clusters, the Z-scored expression values for these genes revealed that the cultivar Gladiator showed stronger responses to the *S. subterranea* infection than Iwa. Moreover, we found 73 genes that were upregulated in the resistant cultivar but downregulated in the susceptible cultivar (Table S1). Functional enrichment analysis for these genes (Table S2) identified several categories related to phenolic polymers and lignin among the most significant functional terms (summarised in Table 1). These included “lignin metabolic process”, “lignin biosynthetic process”, “cinnamyl-alcohol dehydrogenase activity” and “phenylpropanoid metabolic process”. The phenolics polymers and lignin are known to be involved in various disease resistance mechanisms in plants^16–18^. Lignin is one of the most abundant ubiquitous biopolymers and it has been shown that the intermediate phenylpropanoid compounds and metabolites produced during the lignin biosynthesis process are involved in plant defence^19^.

**Table 1:** Enrichment analysis for the genes that upregulated in Gladiator but downregulated in Iwa.

Functional category	Gene count	FDR
ADP binding	9	0.000083
Lignin metabolic process	4	0.00042
Small molecule binding	21	0.00042
Cinnamyl-alcohol dehydrogenase activity	3	0.00042
Nucleotide binding	19	0.00043
Terpene synthase activity	4	0.00099
Carbon–oxygen lyase activity	4	0.0012
Anion binding	19	0.0012
Lignin biosynthetic process	3	0.0014
Phenylpropanoid metabolic process	4	0.0032
Adenyl nucleotide binding	15	0.0032
Ribonucleotide binding	16	0.0032
Adenyl ribonucleotide binding	15	0.0032
Carbohydrate derivative binding	16	0.0032
Phenylpropanoid biosynthetic process	3	0.0059
Secondary metabolic process	4	0.0061
Secondary metabolite biosynthetic process	3	0.0068
Magnesium ion binding	4	0.0067
Purine nucleotide binding	15	0.0067
Carbon–oxygen lyase activity	4	0.0095
Defense response	7	0.0222
UDP-glycosyltransferase activity	5	0.0258
Peptidyl-tyrosine phosphorylation	2	0.0265
Transferase activity, transferring glycosyl groups	6	0.0391

### Resistant and susceptible cultivars exhibited significant differences in GST genes expression

**Figure 2:**
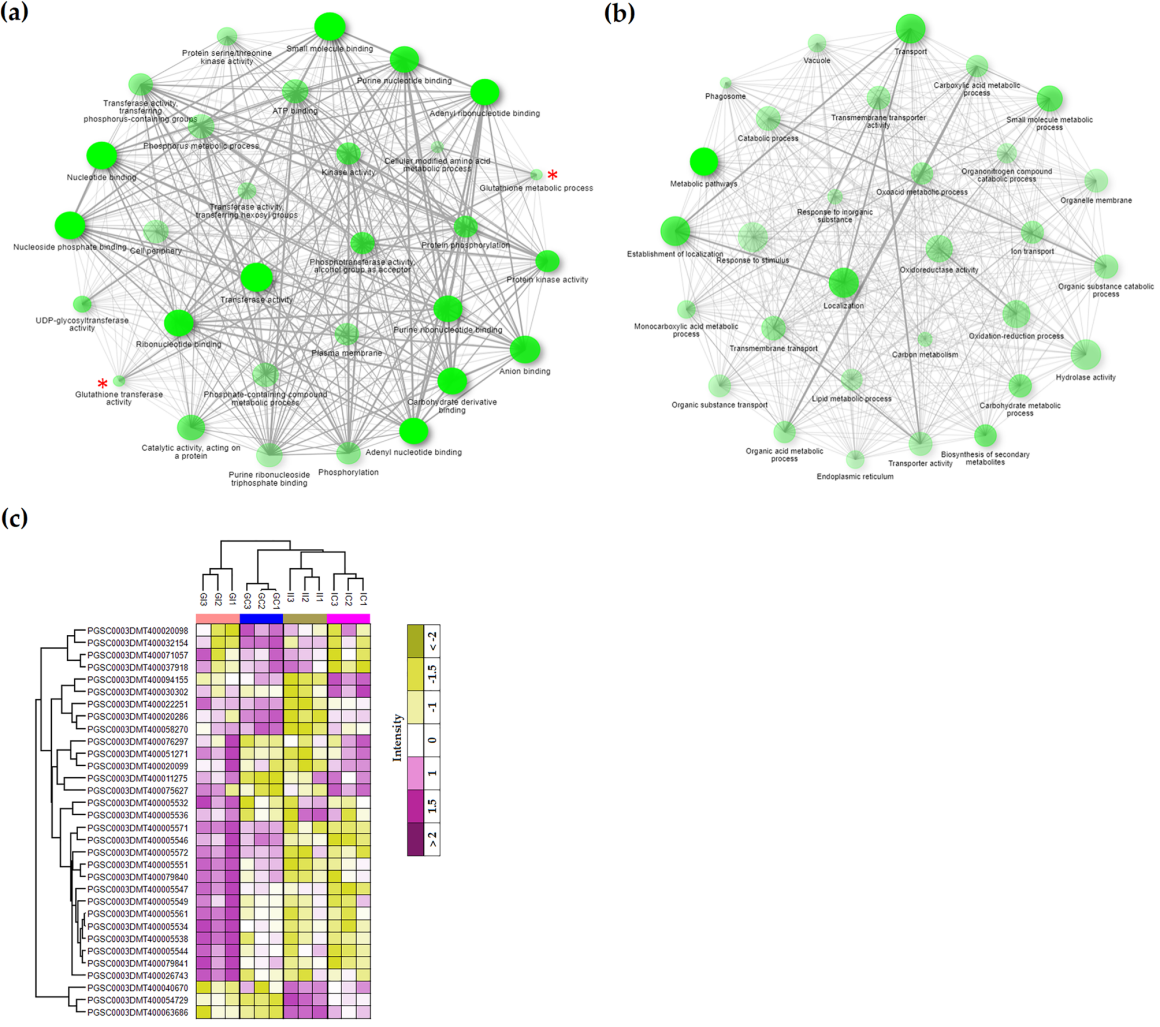
Association networks analysis of the enriched pathway of upregulated DEGs in (**a**) Gladiator and (**b**) Iwa. Darker nodes (more intense in colour) are more significantly enriched gene sets and bigger nodes represent larger gene sets. Thicker edges represent more overlapped genes. The pathways related to “glutathione metabolism” are marked with the red asterisk. (**c**) The Z-scored (normalized) expression of GST genes in the infected and control potato plants. GI, Gladiator infected; GC, Gladiator control; II, Iwa infected; IC, Iwa control.

To further investigate the response to *S. subterranea* infection and identify cellular processes that may play role in pathogen resistance, we used bioinformatics (ShinyGO) to classify genes that were specifically upregulated in Gladiator (n = 848) and those that were specifically upregulated in Iwa (n = 1723) (Table S1). Among the networks of associated significant functional terms in Gladiator (Fig. 2a) or in Iwa (Fig. 2b), two functional clusters were associated with glutathione metabolism (e.g., glutathione metabolic process and glutathione transferase activity). Glutathione S-transferases are ubiquitous enzymes that usually participate in detoxification reactions^20^. The genome-wide analysis of the GST gene family demonstrated the presence of at least 90 GST genes in potato^21^. In the upregulated DEGs in Iwa, several clusters were associated with transport activity but there was no cluster related to glutathione metabolism (Fig. 2b). We then expanded our analyses to test the expression of all GST genes (n = 32) that differentially expressed between infected and uninfected plants. The normalized expression of these genes is presented in a heatmap (Fig. 2c). This analysis revealed that most of the GST genes were upregulated in Gladiator but did not change or were downregulated in Iwa after *S. subterranea* infection. These results suggested that glutathione may play role in resistance against *S. subterranea* root infection^22^.

**Figure 3:**
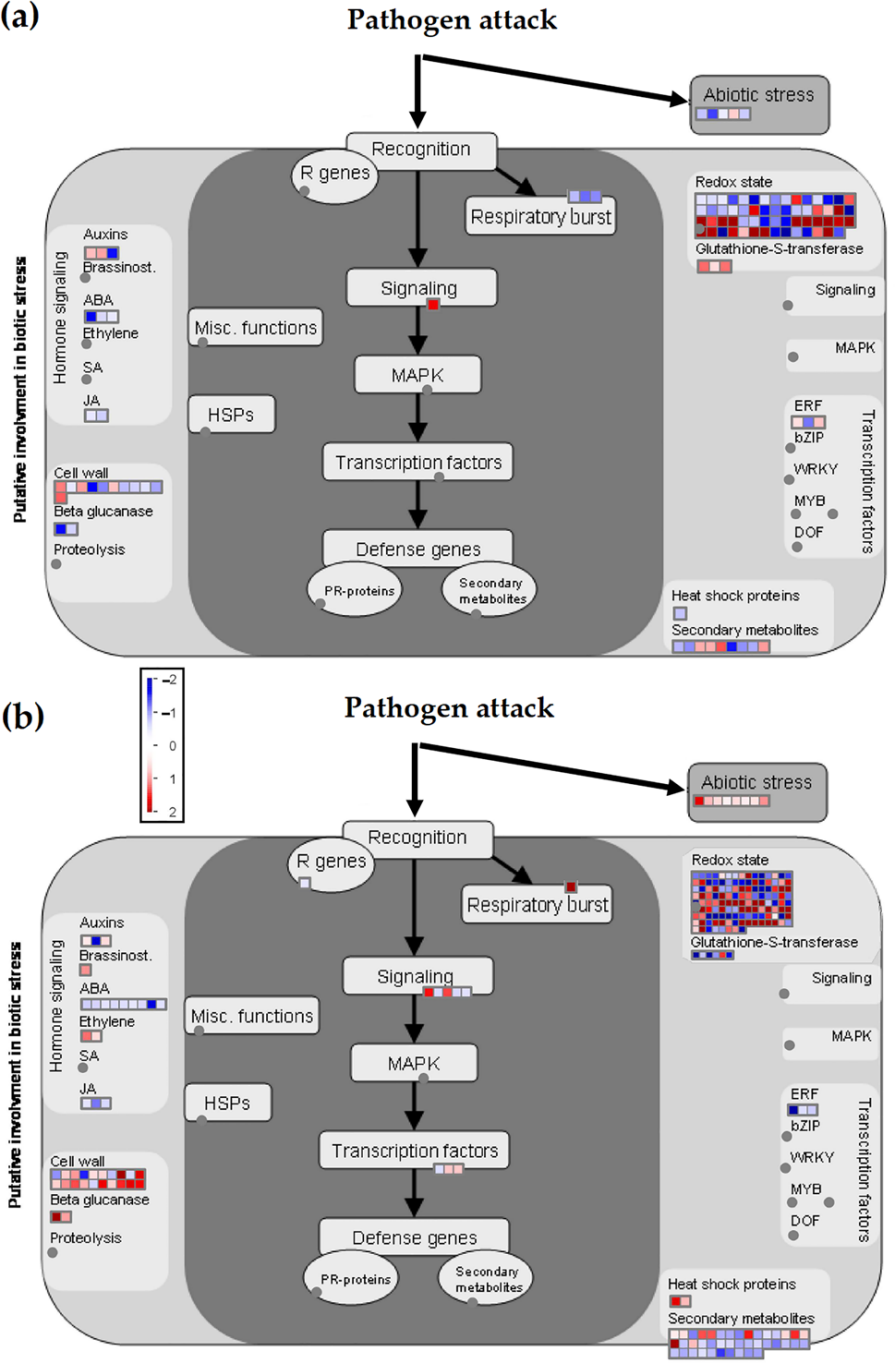
MapMan analysis of changes in biotic stress-associated transcript expression in potato cultivars after 42 days in response to root infection by *S. subterranea*. The log2 fold change of DEGs in (**a**) Gladiator and (**b**) Iwa were mapped to the MapMan biotic stress. The colour scale is shown in middle. A plant’s response to the pathogen attack includes a few steps: recognition of pathogen signal by the related receptors (R genes); induction of the transcription of the cascade of plant immune system including oxidative stress changes; the transition of signals to lead to the production of defense molecules including PR-proteins, secondary metabolites, and heat shock proteins. The big grey circle is an illustrated map of the nucleus, and the small grey circle indicates an annotated biological process. Square blocks represent genes, with up and downregulation marked by red and blue, respectively. Dark grey fields indicate that none of the expressed genes could be assigned to the respective class. ABA, abscisic acid; MAPK, mitogen-activated protein kinase; SA, salicylic acid; JA, jasmonic acid; HSPs, heat shock proteins.

In order to analyse the biotic stress responses after infection, the DEGs in Gladiator (Fig. 3a) and Iwa (Fig. 3b) were mapped to the MapMan biotic stress category. Our data revealed that the most changed processes were redox state, cell wall, secondary metabolism, abiotic stress and Glutathione S-transferases (GSTs). Iwa showed a significant upregulation (n = 8, FDR < 5%) in genes associated with abiotic stress while in Gladiator *S. subterranea* infection did not induce an abiotic stress response. Consistent with the ShinyGO analysis (above), several GST-encoding genes were upregulated in Gladiator, while GST-encoding genes were mainly downregulated in Iwa (Fig. 3).

**Figure 4:**
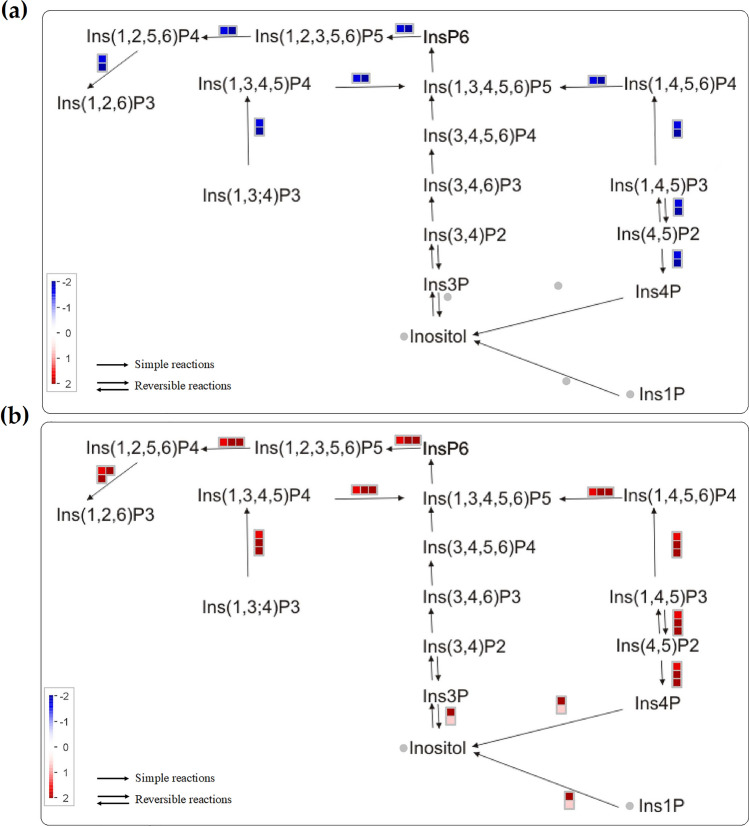
The expression levels of genes from inositol phosphate metabolism among DEGs in (**a**) Gladiator and (**b**) Iwa. Genes are represented by square blocks (expressed as log2 fold change), with up and downregulation are marked by red and blue, respectively. Grey circles indicate that none of the expressed genes could be assigned to the respective class. Ins1P, inositol-1-phosphate; Ins3P, inositol 3-phosphate; Ins4P, inositol-4-phosphate; Ins(1,4,5)P3, inositol 1,4,5-trisphosphate; Ins(4,5)P2, inositol 4,5-bisphosphate; Ins(1,4,5,6)P4, Inositol 1,4,5,6-tetrakisphosphate; Ins(3,4)P2, inositol 3,4-bisphosphate; Ins(3,4,6)P3, inositol 3,4,6-trisphosphate; Ins(3,4,5,6)P4, Inositol 3,4,5,6-tetrakissphosphate; Ins(1,3,4,5,6)P5, Inositol 1,3,4,5,6-pentakisphosphate; Ins(1,3,4)P3, Inositol 1,3,4-trisphosphate; Ins(1,3,4,5)P4, Inositol 1,3,4,5-tetrakisphosphate; InsP6, Myo-inositol hexaphosphate; Ins(1,2,3,5,6)P5, Inositol 1,2,3,5,6-pentaphosphate; Ins(1,2,5,6)P4, Inositol-1,2,5,6-tetraphosphate; Ins(1,2,6)P3, Inositol 1,2,6-trisphosphate.

Inositol phosphate metabolism was one of the biological processes that showed different expression patterns in Gladiator and Iwa. Among the DEGs (FDR < 5%), the expression levels of genes involved in this metabolism were downregulated in Gladiator (Fig. 4a) but were upregulated in Iwa (Fig. 4b). However, the signalling role of the inositol phosphate metabolism in potato is unknown. It has been shown that transgenic Arabidopsis plants that express the human type I inositol polyphosphate 5-phosphatase were more susceptible to *Pseudomonas syringae*^23^. This might explain upregulation of inositol phosphate in the susceptible cultivar after infection.

### Proteomics changes in response to pathogen infection

**Figure 5:**
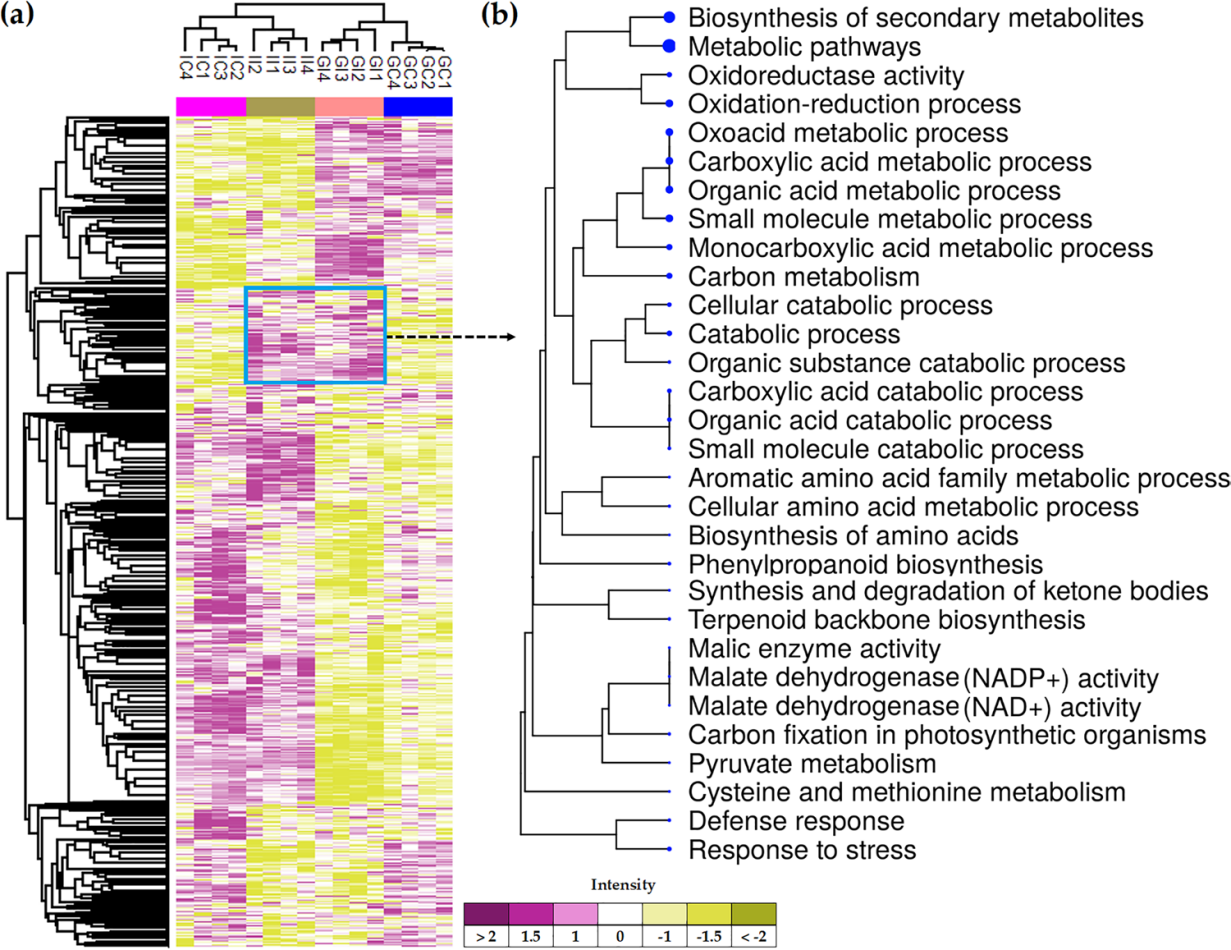
(**a**) The Z-scored (normalized) abundance of the complete set of identified proteins. GI, Gladiator infected; GC, Gladiator control; II, Iwa infected; IC, Iwa control. (**b**) A hierarchical clustering tree summarizing the correlation among significant pathways of the increased DAPs common between Gladiator and Iwa. Pathways with several shared genes are clustered together. Bigger dots (blue dots) indicate more significant *P*-values.

To augment the findings of our transcriptome analysis, we analysed potato roots after inoculation with *S. subterranea*, at the protein level using label-free quantitative proteomics of infected and uninfected plants. In total, 2934 proteins were quantified across both susceptible and resistant cultivars (Table S3). Hierarchical clustering of the identified proteins (total proteins) highlighted a group of proteins that increased abundance in infected plants in both Gladiator and Iwa (Fig. 5a). Functional analysis of these differentially abundant proteins (DAPs) showed that biosynthesis of secondary metabolites and carboxylic acid metabolic processes were the most significant enriched pathways (Fig. 5b). The presence of proteins involved in plant defence and response to stress was also confirmed.

### Major proteomic responses suggested alterations of glutathione metabolism-related proteins in the resistant cultivar

**Figure 6:**
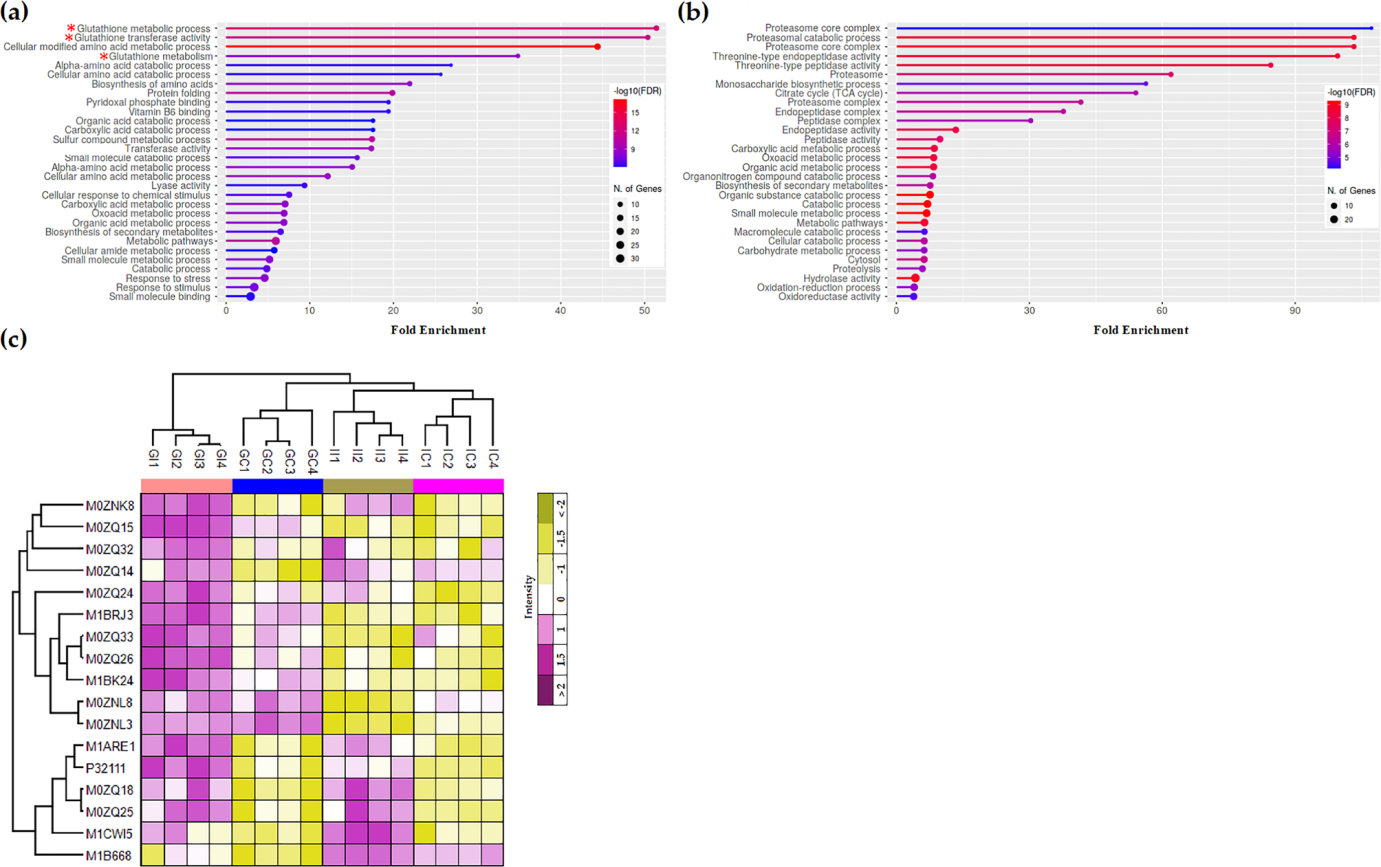
Functional enrichment analysis of the upregulated DAPs in (**a**) Gladiator and (**b**) Iwa. The pathways related to “glutathione metabolism” are marked with the red asterisk. (**c**) The Z-scored (normalized) expression of GST proteins among DAPs in the infected and control potato plants. GI, Gladiator infected; GC, Gladiator control; II, Iwa infected; IC, Iwa control.

We next used the increased DAPs that were found specifically in Gladiator or Iwa (Table S4) to identify cellular processes that might be involved in resistance against *S. subterranea*. The functional enrichment analysis confirmed the presence of three processes related to glutathione metabolism in Gladiator but not in Iwa (Figs. 6 and S4). In Gladiator, “cellular modified amino acid metabolic process”, “glutathione metabolic process” and “glutathione transferase activity” were the most significant enriched processes (Fig. 6a). However, in Iwa, the most significant enriched processes were related to “catabolic process”. In particular, five functional categories associated with proteasome were represented by the proteins in the increased DAPs in Iwa (Fig. 6b). To show the protein abundance patterns for the complete set of GST enzymes we searched for GST proteins that differentially accumulated between infected and uninfected plants. The normalized abondance of 17 GST proteins, found among the DAPs, is presented in a heatmap (Fig. 6c). Except for one GST protein (M1B668), all GST proteins increased in abundance in Gladiator. In contrast, only a few GST proteins increased in abundance in Iwa, while other GSTs were decreased (or unchanged) in abundance after infection (Fig. 6c). In the line with the RNA-seq results, our proteomics data emphasis on the role of glutathione metabolism in resistance against *S. subterreanea*.

### Integration of the proteome and transcriptome

**Figure 7:**
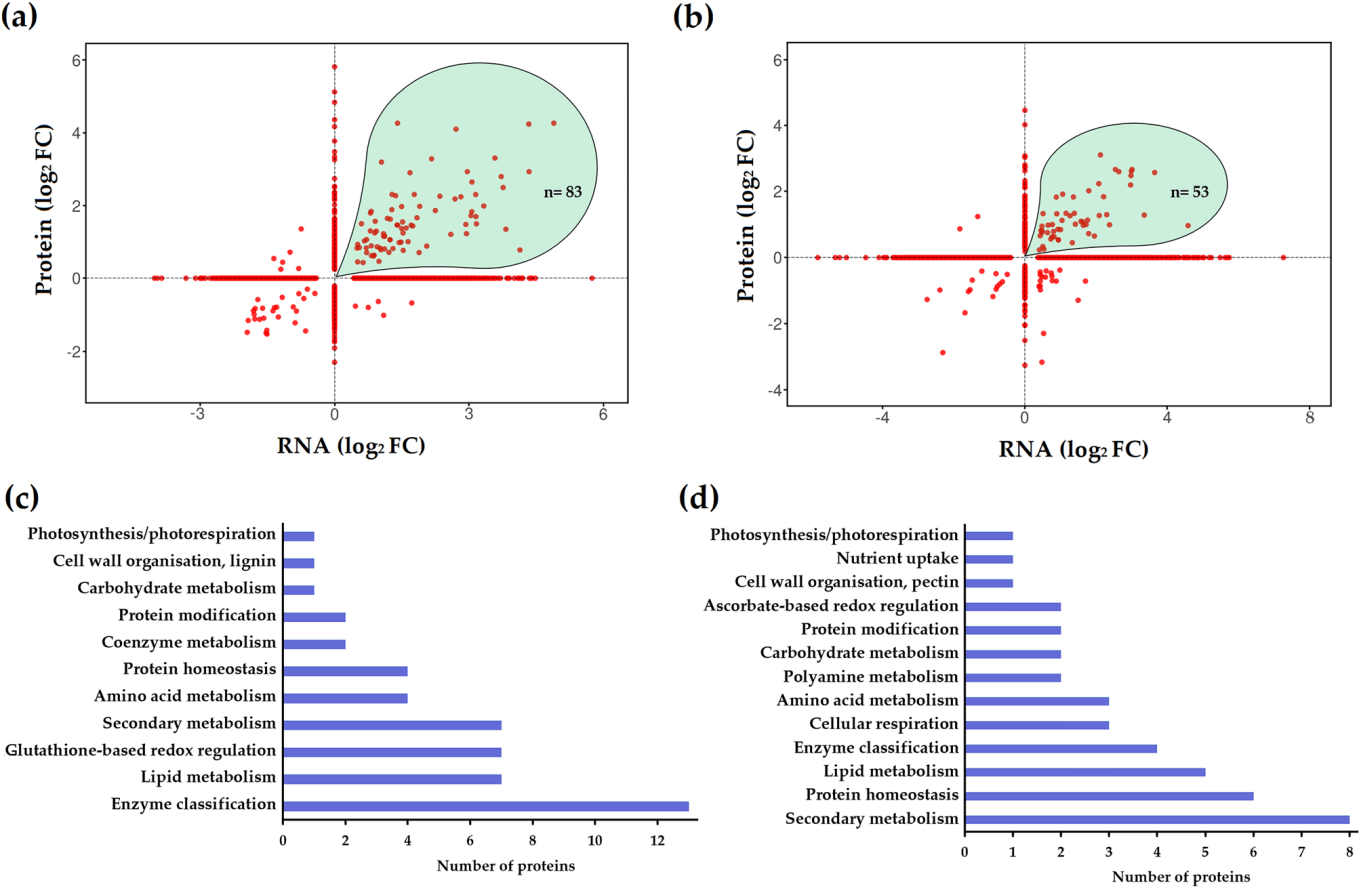
Distribution of the significant proteins common in RNA-seq and proteomics analysis in (**a**) Gladiator and (**b**) Iwa. The highlighted area shows that a total of 83 and 53 genes were significantly upregulated in both RNA and protein levels in Gladiator and Iwa, respectively. Functional annotation of the upregulated proteins in (**c**) Gladiator and (**d**) Iwa.

To integrate proteome and transcriptome data, we focussed on the subset of entities that were significantly altered (FDR < 5%) in at least one of the datasets. The relationship between protein and mRNA fold-changes for Gladiator (n = 2986) and Iwa (n = 4122) is shown in Fig. 7a and b, respectively. This analysis identified 83 proteins and 53 proteins that were upregulated in both RNA-seq and proteome datasets in Gladiator and Iwa, respectively. Functional annotation of these proteins revealed that in Gladiator these proteins were mainly involved in enzyme activity, lipid metabolism, redox homeostasis (glutathione-based redox regulation) and secondary metabolism (Fig. 7c). In Iwa, several of the functional categories overlapped with those in Gladiator, such as protein homeostasis and lipid metabolism (Fig. 7d). Others functions however were cultivar specific including those related to redox homeostasis, providing further evidence for glutathione-based redox regulation in Gladiator.

**Figure 8:**
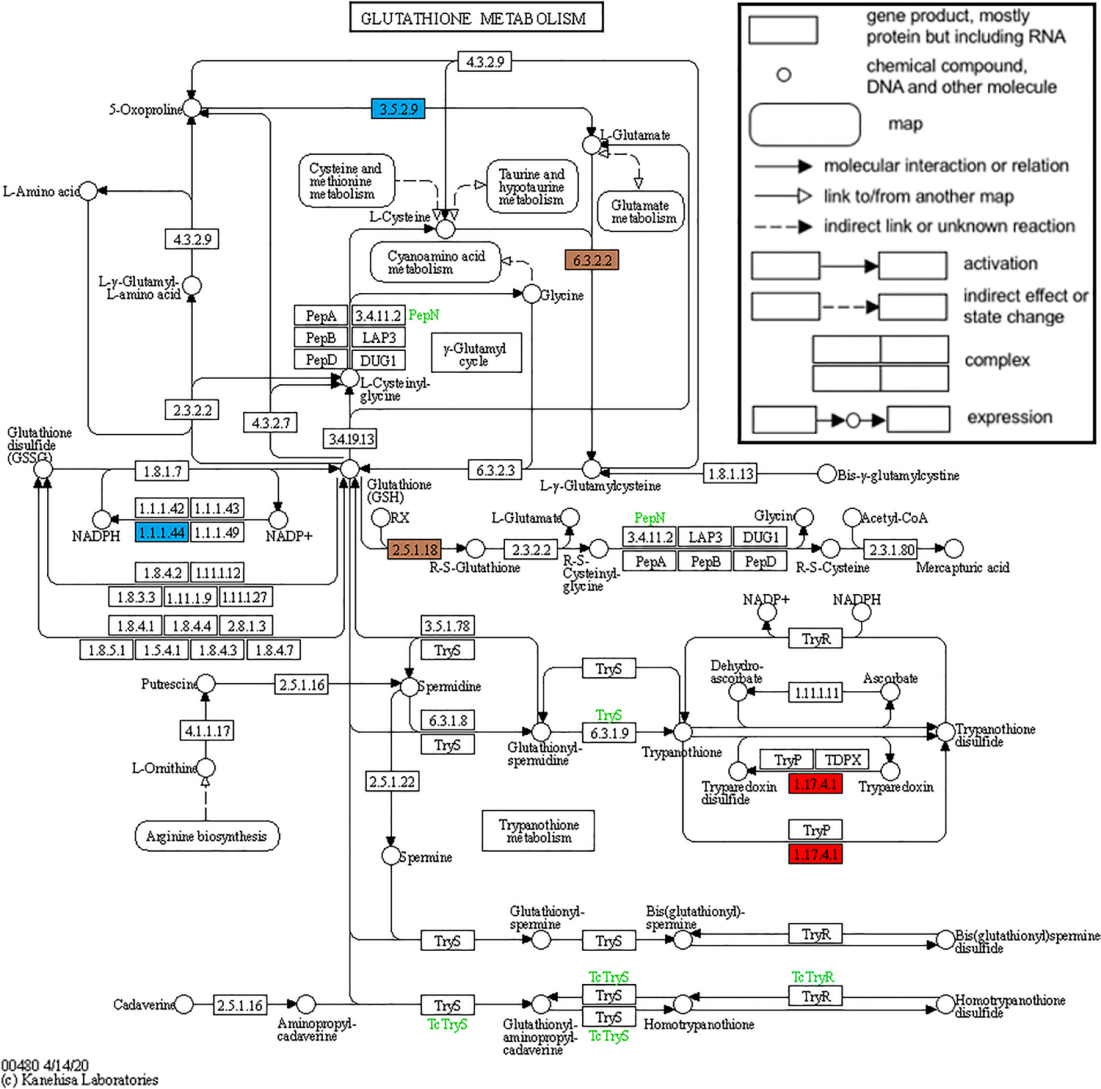
KEGG representation of the Glutathione metabolism (map00480). The upregulated genes and proteins in the resistant cultivar, Gladiator, were mapped to the KEGG glutathione metabolism. KEGG genes products found affected through RNA-seq (red) and proteome analysis (blue) are pinpointed in the figure. The brown squares are representing the genes/proteins that were upregulated in both RNA-seq and proteome analysis. Reprinted from www.genome.jp under a CC BY 4.0 open access license, with permission from Kanehisa Laboratories, original copyright 2022.

The result of our multi-omics analysis highlighted a key role for glutathione metabolism, specifically within the resistant cultivar but not in the susceptible cultivar. We therefore used KEGG pathway analysis to map the genes and proteins significantly upregulated in Gladiator to the glutathione metabolism pathway (Fig. 8). This analysis further highlighted the value of our multi-omics approach by revealing different stages of the glutathione metabolism pathway that were affected after infection by *S. subterranea*. In the RNA-seq data, glutamate-cysteine ligase, glutathione S-transferase and ribonucleoside-diphosphate reductase were the upregulated transcripts. The 5-oxoprolinase, glutamate-cysteine ligase, glutathione S-transferase and 6-phosphogluconate dehydrogenase were the upregulated enzymes in our proteomics data.

## Discussion

This study is the first to examine the molecular response of potato roots to the *S. subterranea* infection at multiple levels. It has been successful in illustrating one mechanism of resistance to disease in potato. It is worth noting that this study should be viewed with an understanding of the complexity of the interaction between a non-culturable obligate biotrophic pathogen and its host plant. Here, we provide new evidence that both glutathione and phenylpropanoid metabolism are strongly associated with resistance to *S. subterranea*. In this section, we discuss the most interesting pathways that were differentially expressed between the resistance and susceptible potato cultivars in response to *S. subterranea* infection.

We observed that the glutathione metabolism was upregulated in the transcriptome and proteome of the resistant cultivar in response to *S. subterranea* infection. To recognise the signals of pathogens, plants utilise a complicated surveillance system. Plants synthesize a diversity of secondary metabolites which play a role in plant defence against pathogens. These metabolites prominently function to protect plants against pathogens according to the toxic nature of pathogens^24^. The timely recognition of the pathogen that enables the rapid deployment of efficient plant defence response is considered as resistance. The susceptibility, however, is a result of weak or late host defense reactions^25^. It has been shown that resistance is often associated with programmed cell death and the accumulation of ROS^8^. Therefore, GSTs that possess glutathione peroxidase activities can be involved in the antioxidative defence against pathogens. Several studies reported that the increase in glutathione-related enzymes is correlated with resistance to different biotic stresses. The decreases in cellular glutathione levels in the susceptible plants can be responsible for pathogen-elicited symptom development^26^. The resistance in tomato against *Oidium neolycopersici*^27^ and oilseed rape against *Sclerotinia sclerotiorum*^28^ were also associated with the upregulation of GSTs. Our proteomics analysis revealed that all identified GSTs were increased in abundance in the resistant cultivar, while we found few GSTs that were decreased in abundance in the susceptible cultivar after *S. subterranea* infection. Similar to our results, proteomic analysis of resistant and susceptible cultivars of oilseed rape showed that proteins involved in the antioxidative defence, including GSTs, only accumulated in the resistant cultivar after infection by *Sclerotinia sclerotiorum*^29^. The overexpression of a GST gene (OsGSTU5) in rice increased the resistance against a necrotrophic fungus, *Rhizoctonia solani*, whereas the knockdown line had greater lesion coverage, hyphal penetration and pathogen transcript level^30^.

In our transcriptome analysis, we found 73 genes that were upregulated in Gladiator but downregulated in Iwa. The enrichment analysis demonstrated that these genes were mostly involved in lignin biosynthesis. In the proteome analysis, the proteins related to the cell wall were differentially expressed in two cultivars after infection. For example, caffeic acid (5-hydroxyconiferaldehyde) O-methyltransferase (COMT) which is one of the enzymes involved in the lignin biosynthesis pathway did not change in Gladiator but was decreased in abundance in Iwa after infection. Several studies have indicated that numerous genes associated with cell wall organization are involved in plant defence against pathogens^31–34^. Therefore, it is believed that the phenylpropanoid metabolic process and in particular lignin biosynthesis contribute to resistance against pathogens in plants^33^. The establishment of mechanical barriers to pathogen invasion, restricting polysaccharide degradation by pathogen enzymes and limiting the diffusion of toxins and nutrients from the pathogen to the host is the proposed mechanism behind the resistance associated with the phenylpropanoid metabolic process^35^. Together with these results, our findings in transcriptome and proteome analysis of potato root infected by *S. subterranea* suggest that cell wall proteins play a crucial role in resistance to *S. subterranea*.

The upregulation of the inositol phosphate pathway in the susceptible cultivar was observed in the DEGs . The involvement of several lipids in the interaction between pathogens and their host plants has been previously confirmed^36^. However, there is less information on the potential involvement of inositol phosphates in the plant-pathogen interaction. Gonorazky, et al.^37^ showed that silencing of the tomato phosphatidylinositol-phospholipase C2 reduced the plant susceptibility to *Botrytis cinerea*, while expression of the human type I inositol polyphosphate 5-phosphatase increased susceptibility of Arabidopsis to *Pseudomonas syringae*^23^. One of the earliest responses triggered by the recognition of MAMPs in plants is the activation of phosphoinositide-specific phospholipase. The phosphoinositide-silenced plants had reduced ROS-dependent responses such as cell wall re-organisation (e. g. callose deposition) in response to microbial infection^38^. In the line with these studies, our results confirmed the upregulation of inositol phosphate in the susceptible cultivar; Iwa. Therefore, we conclude that the upregulation of the inositol phosphate pathway might cause potato susceptibility to *S. subterranea* infection by reducing lignification processes and ROS-dependent responses.

In Iwa, five functional categories associated with proteasome were represented by the proteins in the increased DAPs. The ubiquitin proteasome system (UPS) plays a central role in protein degradation processes in plants and has been identified as an important component of plant-pathogen interactions in several contexts^39^. Regardless of pathogen type, the UPS is involved in several steps of plant defence. However, the UPS is not only used by plants to protect themselves, it can also be used by some pathogens for their own purposes^40^. For instance, *Pseudomonas syringae* (which does not have an endogenous UPS) targeted host proteins for degradation by harnessing the host UPS^41,42^.

In summary, our study provides the first multi-omics data of *Spongospora*-potato interaction, adding important pieces to our understanding of the mechanisms of resistance to *S. subterranea*. The upregulation of glutathione metabolism in the resistant cultivar in both RNA and protein levels is one of the key findings of the present study. This pathway plays a critical role in the plant redox and thereby mediates immune signalling. The involvement of inositol phosphate in the susceptivity to *S. subterranea* was also confirmed in the RNA-seq and proteomics analysis. Future work will aim to uncover the details surrounding the interplay between the inositol phosphate signalling and the plant immune system. More than that, the study provides databases for meta studies and can allow the exploitation of this knowledge for the benefit of agriculture.

## Experimental procedures

### Plant materials and pathogen infection

We acknowledge the use of plant materials in this manuscript complies with all relevant institutional, national, and international guidelines and legislation. Potato cultivars ‘Gladiator’ and ‘Iwa’ used in this study were kindly supplied by NZ Plant and Food Research (variety owners). Cultivar ‘Iwa’ is known as highly susceptible to both tuber and root disease, while cultivar ‘Gladiator’ has a strong resistance to both root and tuber infection by *S. subterranea*^43^. The plants were maintained in tissue culture at 22 °C under a 16 h light/8 h dark photoperiod for three weeks in a basic Murashige & Skoog (MS) medium with 500 mg/L of casein hydrolysate, 30 g/L sucrose and 40 mg/L of ascorbic acid was used as the growth medium. Dried *S. subterranea* inoculum was obtained from powdery scab-infected tubers as previously described^44^. To stimulate germination of the resting spores, two milligrams of dried *S. subterranea* resting spores (approximately 20,000 sporosori per mg) were suspended in 2 mL of Hoagland solution^45^ and incubated at 25 °C for 72 h. Ten tissue cultured plantlets of each cultivar of similar size (~ 8 cm) were inoculated by suspending their roots into the inoculum suspension for one hour. A similar number of uninoculated plants of each cultivar were suspended in sterile water only for one hour. The seedlings were then transplanted into 2L plastic pots filled with sterilised potting mix (sand, loam, composted pine bark). The plants were maintained under controlled conditions in the greenhouse (25 ± 3 °C, 16 h photoperiod, 80 ± 5% humidity). Two weeks after planting, an additional 20 mL of fresh inoculum suspension was added to each pot, to ensure ongoing disease pressure. After 42 days in the greenhouse, plants were harvested and the roots of the infected and control plants were collected, washed thoroughly under running water, frozen in liquid nitrogen and stored at -80 °C for RNA-seq (n = 3) and proteomics (n = 4) experiments.

### RNA extraction, sequencing and transcriptome assembly

Total RNA was extracted from 50 mg of frozen roots from three biological replicate plants per condition using the RNeasy plus mini kit (Qiagen, Hilden, Germany). Contaminant genomic DNA was removed from the samples using the gDNA Eliminator spin columns from the RNeasy plus mini kit. The quantity and quality of total RNA were checked by a Qubit fluorometer (Invitrogen, Waltham, MA, USA) and an Agilent 2100 Bioanalyzer system (Agilent, Palo Alto, Santa Clara, CA, USA), respectively. Total RNA was then used to construct mRNA-seq libraries using TruSeq Stranded Total RNA Library Prep Kit with RiboZero (Illumina, San Diego, CA, USA) and sequenced at the Australian Genome Research Facility (AGRF, Melbourne, Victoria, Australia) to generate > 50 million (paired-end) 100-nucleotide NovaSeq6000 Illumina reads per sample. After quality checks using FastQC (available on the Galaxy Australia platform; https://usegalaxy.org.au/), the adapter contaminations were trimmed from the reads by Trimmomatic^46^. The high-quality trimmed RNA-seq reads were then mapped to the potato genome (http://spuddb.uga.edu) using HISAT2^47^ with default parameters. The uniquely mapped reads were determined using a count matrix of mapped fragments per reference-sequence annotated gene, generated by FeatureCounts^48^. To identify the differentially expressed genes (DEGs), we used DESeq2^49^ with a False Discovery Rate (FDR)-adjusted *P*-value cut-off set to 0.05.

### Protein extraction

The frozen roots (50 mg per sample) were homogenised in 150 µL of extraction buffer (7 M urea and 2 M thiourea, 100 mM NaCl, protease inhibitor cocktail (cOmplete Mini EDTA-free; Roche Diagnostics, NSW, Australia), 1% dithiothreitol (DTT), and 40 mM Tris, pH 8.0) using a Fast Prep-24 bead beater (Mp Biomedicals, Seven Hills, NSW, Australia) for 60 s. Extracts were then centrifuged at 16,000 g in the cold room (4 °C) for 10 min. The collected supernatant was added to six volumes of absolute acetone chilled to − 20 °C and kept at − 20 °C overnight. To concentrate precipitated protein, samples were centrifuged at 10,000* g* for 8 min. The protein pellet obtained was washed using cold acetone and left to air dry to remove any acetone residue. The pellet was then resuspended in denaturing buffer (7 M urea and 2 M thiourea, protease inhibitor and 40 mM Tris, pH 8.0). Protein concentrations were calculated using a Qubit fluorometer (Thermo Fisher Scientific, Waltham, MA, USA). Proteins were reduced overnight at 4 °C by adding DTT in a final concentration of 10 mM. For the alkylation, 50 mM iodoacetamide was added to the samples and incubated for 2 h at room temperature in dark. Proteins (30 µg per sample) were then digested according to the standard SP3 method^50^ using trypsin/LysC (Promega, Madison, WI, USA). Peptides were dried down in the SpeedVac concentrator after desalted using ZipTips (Merck, Darmstadt, Germany).

### LC–MS/MS analysis and label-free quantification

The dried peptides were reconstituted in 12 µL of HPLC loading buffer (2% acetonitrile and 0.05% TFA in water). Peptides (~ 1 µg per sample) were analysed by nanoLC-MS/MS using a Q-Exactive HF and Ultimate 3000 nanoHPLC system (ThermoFisher Scientific, Waltham, MA, USA). Tryptic peptides were loaded onto a 20 mm PepMap 100 C18 trapping column (3 mm C18) at 5 µL min^−1^. Peptides were separated at 300 nl min^−1^ on a 250 mm PepMap 100 C18 analytical column at 45 °C using a two-hour segmented gradient from mobile phase A (0.1% formic acid in water) to mobile phase B (0.08% formic acid in acetonitrile/water (80:20)). The separation phase comprised sequential increase in % mobile phase B from 2 to 10% over 7 min, 10 to 25% over 68 min, 25 to 45% over 21 min followed by column washing in 95% B and re-equilibration in 2% B. The mass spectrometer was controlled by Xcalibur 4.3 in a data-independent acquisition (DIA) mode. MS1 spectra were acquired in profile mode at 120,000 resolution (scan range 390–1240 m/*z*) while MS2 spectra of 26 × 25 amu sequential windows(scan range 402.5–1027.5 m/*z*, 1 amu overlap) were collected in centroid mode at 30,000 resolution. RAW files were processed using Spectronaut software (v 14.7) whereby the DIA-MS spectra were used to generate a spectral library using the Pulsar search engine and the FASTA file of 53,106 entries comprising the UniProt proteome for *Solanum tuberosum*. Further details of Xcalibur and Spectronaut software parameter setting are described in Balotf et al.^51^. Proteins identified on the basis of a single matching peptide were excluded. Downstream data analysis for the proteomics data was performed using Perseus version 1.6.14.0^52^. Data were first log_2_-transformed, and proteins with fewer than four valid values in any of the four treatment groups were removed from the dataset. Remaining missing values were imputed according to Perseus default settings.

### Bioinformatic analysis

Association networks were constructed using ShinyGO^53^ and String databases (https://string-db.org/). We used the MapMan tool^54^ to display our large datasets (RNA-seq and proteomics) onto diagrams of the metabolic pathways. MapMan version 3.5.1 was loaded with *S. tuberosum* data downloaded from the MapManStore server (http://mapman.gabipd.org/web/guest/mapmanstore). Our listing IDs for both protein and RNA data were loaded into MapMan after log2-transformed conversion along with fold change (FC) values. A colour scheme with a scale level that allowed an easy visualization of upregulated (red) or downregulated (blue) proteins or genes was used for all MapMan diagrams. Mercator4 (https://plabipd.de/portal/mercator4), DAVID bioinformatics resources^55^ and Kyoto Encyclopedia of Genes and Genomes (KEGG)^56^ were used to identify representative functional networks and metabolic pathways for the proteomics and RNA-seq data. Heatmaps were drawn using Perseus software, Principal component analysis (PCA) plots were obtained from MetaboAnalyst (https://www.metaboanalyst.ca) and the volcano plot was obtained from the VolcaNoseR app^57^.

## Supplementary Information

Supplementary Information 1.

Supplementary Information 2.

Supplementary Information 3.

Supplementary Information 4.

Supplementary Information 5.

Supplementary Information 6.

Supplementary Information 7.

Supplementary Information 8.

## Data Availability

The RNA-seq raw reads are available via the NCBI-SRA database under BioProject PRJNA776331. The MS/MS raw data were deposited to the ProteomeXchange Consortium via the PRIDE^58^ partner repository with the dataset identifier PXD029381.
