# Supplementary material for: Multi-omics reveals mechanisms of resistance to potato root infection by Spongospora subterranea
Source: Sci Rep. 2022 Jun 25;12:10804. doi: 10.1038/s41598-022-14606-y (PMC9233701; doi:10.1038/s41598-022-14606-y)

**Fig. S1**

Root gall formation in Gladiator (left) and Iwa (right). Both cultivars developed root galls 42 days after infection (indicated with a red circle in the picture). However, the number of galls, as well as their size, was bigger in Iwa.

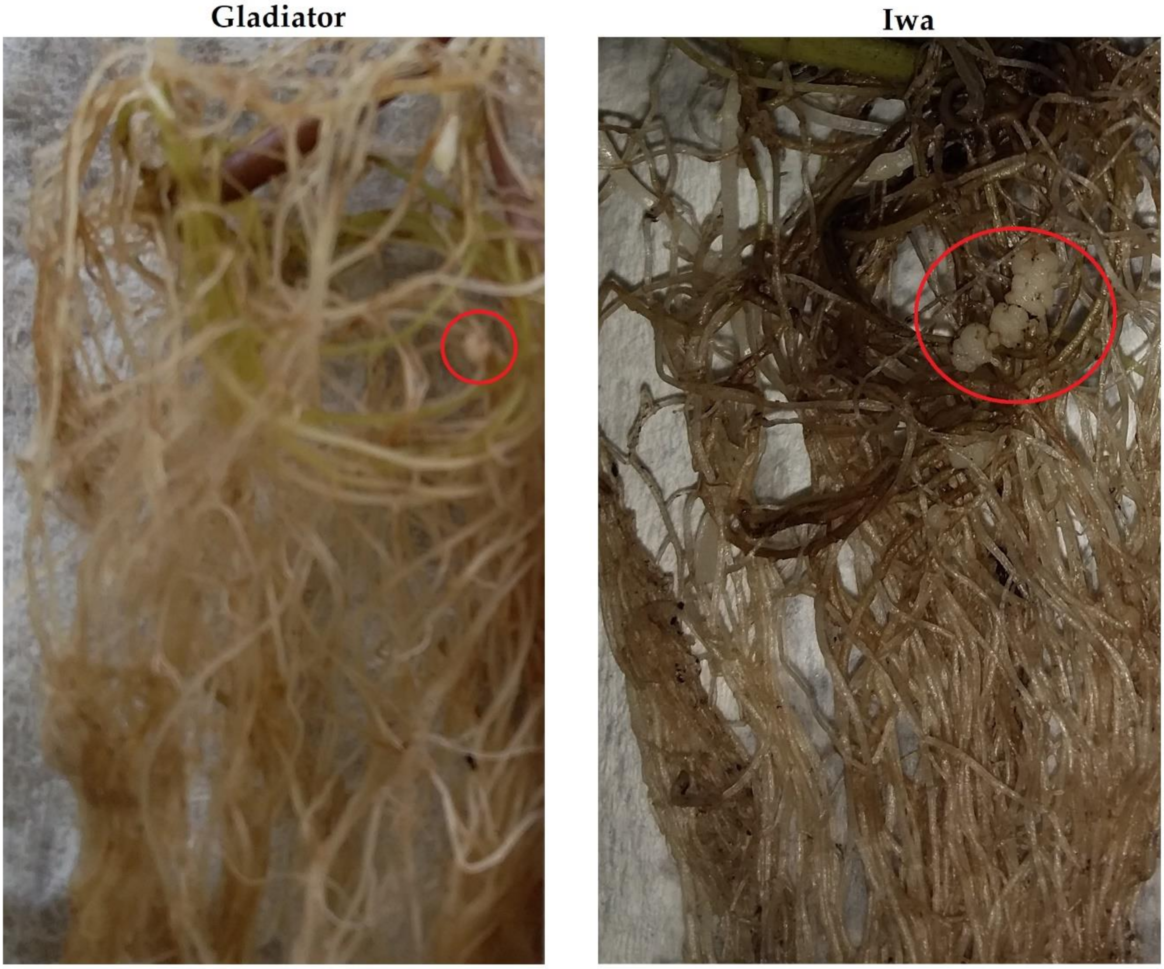

Supplement: Supplementary file 1 — Supplementary Information 1. [file 41598_2022_14606_MOESM1_ESM.pdf]
