# Supplementary figures and images for: Multi-omics reveals mechanisms of resistance to potato root infection by Spongospora subterranea
Source: Sci Rep. 2022 Jun 25;12:10804. doi: 10.1038/s41598-022-14606-y (PMC9233701; doi:10.1038/s41598-022-14606-y)

**Fig. S2.** RNA-seq alignment statistics

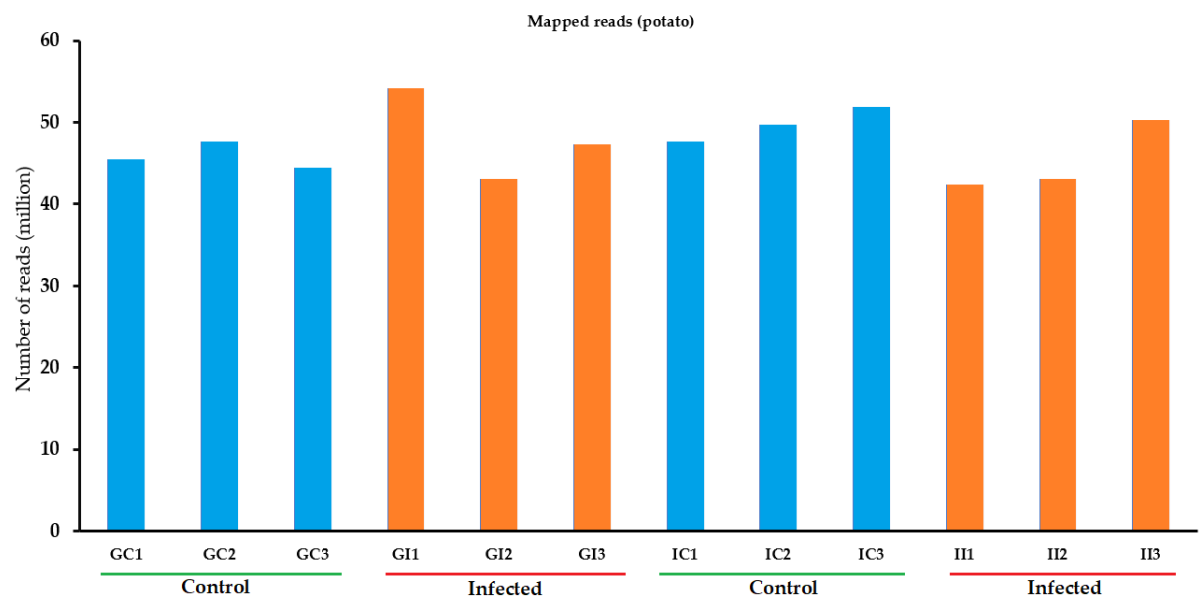

Supplement: Supplementary file 2 — Supplementary Information 2. [file 41598_2022_14606_MOESM2_ESM.pdf]

(a)

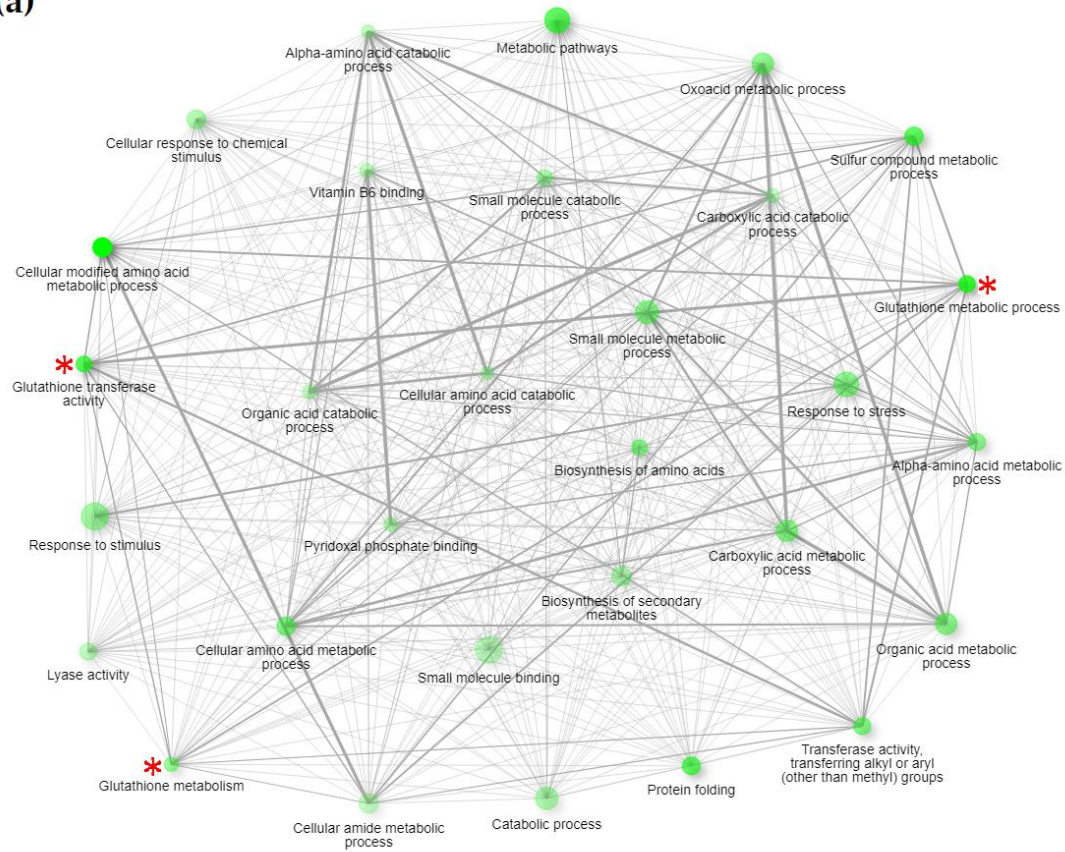

(b)

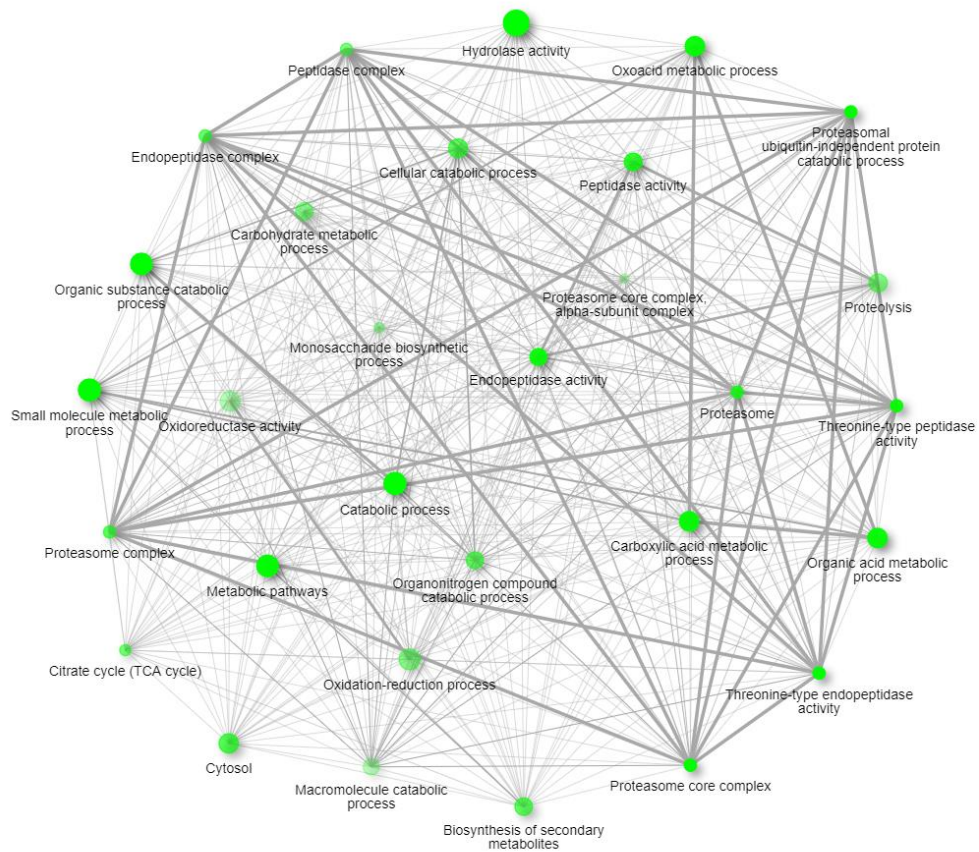

Supplement: Supplementary file 4 — Supplementary Information 4. [file 41598_2022_14606_MOESM4_ESM.pdf]
