# Supplementary material for: Multi-omics reveals mechanisms of resistance to potato root infection by Spongospora subterranea
Source: Sci Rep. 2022 Jun 25;12:10804. doi: 10.1038/s41598-022-14606-y (PMC9233701; doi:10.1038/s41598-022-14606-y)

Fig S3. Differentially expressed genes (FDR < 5%) regulated by pathogen inoculation in the resistant (Gladiator) and susceptible (Iwa) cultivars. GI; Gladiator infected, GC; Gladiator control, II; Iwa infected; IC; Iwa control.

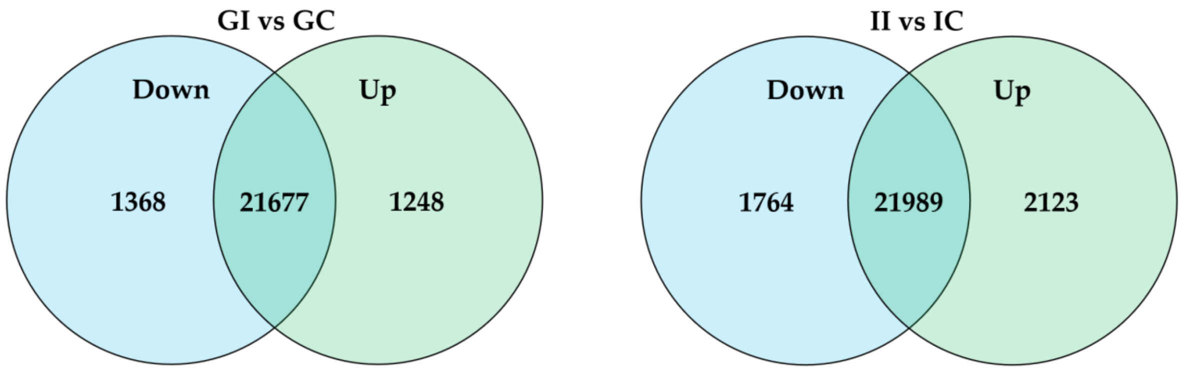

Supplement: Supplementary file 3 — Supplementary Information 3. [file 41598_2022_14606_MOESM3_ESM.pdf]
